# Efficacy of Spice Supplementation in Rheumatoid Arthritis: A Systematic Literature Review

**DOI:** 10.3390/nu12123800

**Published:** 2020-12-11

**Authors:** Jean-Guillaume Letarouilly, Pauline Sanchez, Yann Nguyen, Johanna Sigaux, Sébastien Czernichow, René-Marc Flipo, Jérémie Sellam, Claire Daïen

**Affiliations:** 1 Department of Rheumatology, CHU Lille, Université de Lille, F-59000 Lille, France; jeanguillaume.letarouilly@chru-lille.fr (J.-G.L.); rene-marc.flipo@chru-lille.fr (R.-M.F.); 2 Department of Rheumatology, CHU de Montpellier, Montpellier University, 34295 Montpellier, France; pauline.sanchez93@gmail.com; 3 Department of Internal Medicine, Hôpital Beaujon, AP-HP Nord, Université de Paris, F-92100 Clichy, France; yann.nguyen2@aphp.fr; 4 Department of Rheumatology, Hôpital Avicenne, AP-HP, INSERM U1125, Université Paris 13, F-93017 Bobigny, France; johanna.sigaux@aphp.fr; 5 Department of Nutrition, Specialized Obesity Center, Hôpital Européen Georges Pompidou, Université de Paris, AP-HP, F-75015 Paris, France; sebastien.czernichow@aphp.fr; 6 Epidemiology and Biostatistics Sorbonne Paris City Center, UMR1153, Institut National de la Santé et de la Recherche Médicale, F-75004 Paris, France; 7 DMU 3ID, Hôpital Saint Antoine, AP-HP, CRSA Inserm UMRS_938, Sorbonne Université, F-75012 Paris, France; jeremie.sellam@aphp.fr

**Keywords:** rheumatoid arthritis, spice, garlic, cinnamon, curcuma, saffron, ginger, disease activity

## Abstract

Background: Spices, i.e., curcumin, ginger, saffron, and cinnamon, have a thousand-year history of medicinal use in Asia. Modern medicine has begun to explore their therapeutic properties during the last few decades. We aimed to perform a systematic literature review (SLR) of randomized controlled trials (RCTs) assessing the effect of spice supplementation on symptoms and disease activity in patients with chronic inflammatory rheumatic diseases (rheumatoid arthritis (RA), spondylarthritis, or psoriatic arthritis). Methods: An SLR of RCTs, reviews, and meta-analyses was performed, searching for articles in MEDLINE/PubMed. Abstracts from international rheumatology and nutrition congresses (2017–2020) were also scrutinized. The risk of bias of the selected studies was evaluated using the Cochrane Collaboration’s tool and the Jadad scale. Results: Altogether, six studies, assessing the use of spice supplementation only in RA patients, were included: one on garlic supplementation, two on curcumin, one on ginger, one on cinnamon, and one on saffron supplementation. Garlic, ginger, cinnamon, or saffron supplementation was associated with a decrease in RA clinical activity. However, several points limit the external validity of these studies. No conclusion on the impact of curcumin supplementation on RA activity could be drawn due to low-quality studies. Conclusions: Garlic, ginger, cinnamon, and saffron supplementation could have a beneficial effect on RA activity, but the risk of bias of these studies is difficult to assess and data are too limited to recommend them in daily practice.

## 1. Introduction

Spices are defined by the Food and Drug Administration organization (FDA) as “aromatic vegetable substances, in the whole, broken, or ground form, whose significant function in food is seasoning rather than nutrition” [1]. Spices have played important roles as flavoring agents, food preservatives, and medicines [2]. Spices have a thousand-year history of medicinal use in Asia. For example, turmeric which contains curcumin has been used in India and is called the “golden spice” due to its brilliant yellow color. It is mentioned as a treatment in the 250 Before Common Era (BCE) Ayurvedic treatise [3]. Ginger has been produced by the Indian and Chinese for over 5000 years. It is an important ingredient in Chinese, Ayurvedic, and Tibb-Unani medicines for the treatment of catarrh, rheumatism, nervous diseases, gingivitis, toothache, asthma, stroke, constipation, and diabetes [4]. Modern medicine has begun to explore their medical potential during the last few decades. There is an increasing number of published articles assessing the health potential of spices in PubMed: from 17 in 1966 to 3407 in 2020.

The assessment of their health benefits has increased significantly, as many spices are known to possess properties associated with reducing the risk of developing chronic diseases [1]. Several spices have known or potential anti-inflammatory activity, mostly by inhibiting the nuclear factor (NF)-κB pathway or cyclooxygenase (COX) activation [5,6,7].

Given their known and potential health benefits, spices could be appealing to patients with chronic inflammatory rheumatic diseases as a natural complement to their disease-modifying antirheumatic drugs (DMARDs).

The objective of this systematic literature review (SLR) was to summarize data of randomized controlled trials assessing spice supplementation to reduce inflammatory rheumatic disease symptoms and activity. This SLR was used to inform the recommendations of the French society of Rheumatology on diet in inflammatory rheumatic diseases.

## 2. Materials and Methods

This systematic literature review of randomized controlled trials (RCTs), reviews, and meta-analyses was performed according to PRISMA (Preferred Reporting Items for Systematic Reviews and Meta-Analyses) recommendations [8].

### 2.1. Eligibility Criteria

Studies considered eligible were as follows: (1) open-label or double-blind randomized controlled studies, systematic literature reviews, and meta-analyses, (2) including patients with confirmed inflammatory rheumatic diseases (rheumatoid arthritis (RA), psoriatic arthritis (PsA), or spondylarthritis (SpA)), (3) having evaluated the use of oral supplementation of spices (garlic, cinnamon, curcuma, saffron, or ginger) (4) with a control group (5) on symptoms and disease activity. Cutaneous administrations of spices were excluded. Disagreement in the determination of the eligibility of each study was resolved by consensus.

### 2.2. Search Strategy

MEDLINE (Via PubMed) databases were used to search for potentially eligible articles, from inception until June 2020, in English and French.

Original research papers and reviews were searched using combinations of the grouped search terms: (“Spondylitis, Ankylosing” [Mesh] OR ankylosis OR Spondylarthritis OR Spondylarthropathies OR “Spondylarthritis” [Mesh] OR “Spondylarthritides” OR “Spinal Arthritis” OR “Spinal Arthritides” OR “Arthritis, Spinal” OR “Spondyloarthritis” OR “Arthritis, Rheumatoid” [Mesh] OR “rheumatoid arthritis” OR “rheumatoid” OR “Caplan Syndrome” OR “Felty Syndrome” OR “Rheumatoid Nodule” OR “Rheumatoid Vasculitis” OR “Arthritis, Psoriatic” [Mesh] OR “Psoriasis” OR “Arthritic Psoriasis” OR “Psoriatic Arthritis” OR “Psoriasis Arthropathica” OR “Psoriatic Arthropathy” OR “Arthropathies, Psoriatic” OR “Arthropathy, Psoriatic” OR “Psoriatic Arthropathies”) AND (“Spices” [Mesh] OR “Spice” OR “Spices” OR “Garlic” [Mesh] OR “Allium” [Mesh] OR “Allium sativum” OR “garlic” OR “Curcuma” [Mesh] AND “Curcumas” OR “Tumeric” OR “Tumerics” OR “Turmeric” OR “Turmerics” OR “Curcuma zedoaria” OR “Curcuma zedoaries” OR “zedoaria, Curcuma” OR “Zedoary zedoaria” OR “Zedoary zedoaries” OR “zedoaria, Zedoary” OR “Curcuma longa” OR “Curcuma longas” OR “longa, Curcuma” OR “Ginger” [Mesh] OR “Gingers” OR “Zingiber officinale” OR “Zingiber officinales” OR “officinales Zingiber” OR “Cinnamomum zeylanicum” [Mesh] OR “Cinnamomum verum” OR “Cinnamon” OR “Cinnamons” OR “Cinnamomum” [Mesh] OR “Cinnamomums” OR “Crocus” [Mesh] OR “Saffron” OR “Saffrons” OR “Crocus sativus” OR “Saffron Crocus” OR “Crocus, Saffron” OR “Iridaceae” [Mesh]).

Conference abstracts from international Rheumatology meetings (European League against Rheumatism (EULAR), Société Française de Rhumatologie (SFR), and the American College of Rheumatology (ACR)) and from Nutrition meetings (International Congress of Nutrition, European Nutrition Conference, American Society of Nutrition) from 2017 to 2020 were also screened with keywords “spice”, “garlic”, “cinnamon”, “curcuma/curcumin”, “saffron”, “ginger”, or “rheumatoid arthritis” and then manually reviewed for inclusion. A manual search based on references of the selected articles was performed.

### 2.3. Data Extraction

A data extraction table was used to extract data of the eligible studies and to systematically describe study characteristics, such as study design, aim, population, eligibility criteria, sample size, intervention, primary and secondary assessed outcome, side effects, and treatment adherence.

### 2.4. Quality Assessment

Risk of bias of each study was assessed using the Cochrane Collaboration’s tool for assessing risk of bias [9] and the Jadad scale [10]. Records limited to abstracts were not assessed because of the paucity of available information.

## 3. Results

### 3.1. Study Selection

**Figure 1:**
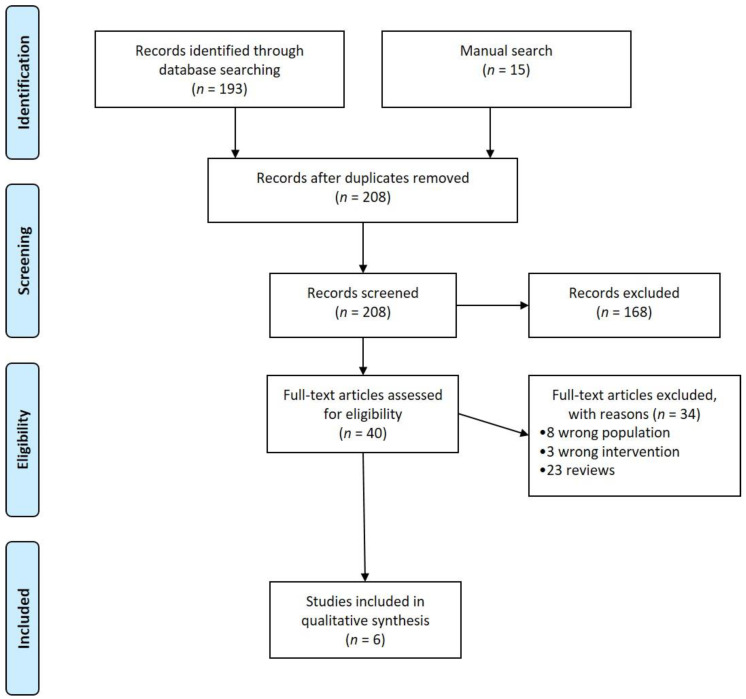
Preferred Reporting Items for Systematic Reviews and Meta-analyses (PRISMA) diagram.

A total of 208 records were identified in the search process (Figure 1). Of these, six were selected after review and assessed for eligibility. No relevant unpublished studies from rheumatology and nutrition congresses were obtained.

### 3.2. Study Characteristics

The study characteristics of the selected studies are summarized in Table 1. All studies assessed the impact of spice supplementation on patients with RA: one assessed garlic supplementation, two assessed curcumin, one assessed ginger, one assessed cinnamon, and one assessed saffron supplementation. No randomized controlled trials were found regarding PsA or SpA patients. All selected studies were published in English. Follow-up duration ranged from 8 to 12 weeks.

Studies’ interventions and outcomes are reported in Table 2. The six studies assessed the efficacy on spice supplementation in reducing RA activity on patients with active RA, using clinical activity indices (disease activity score (DAS)-28 or ACR response), health assessment questionnaire (HAQ), number of tender or swollen joints, pain visual analogue scale (VAS), disease activity analogue scale, and morning stiffness duration. C reactive protein (CRP) and erythrocyte sedimentation rate (ESR) were also used.

The included studies involved 316 RA patients. The main inclusion criteria were participants with active RA diagnosed according to the 1987 ACR criteria or 2010 ACR EULAR criteria. The comparator was placebo in all studies except one (Chandran et al.) in which curcumin was compared to diclofenac. The outcomes were assessed at 8 or 12 weeks.

### 3.3. Risk of Bias within Studies

**Figure 2:**
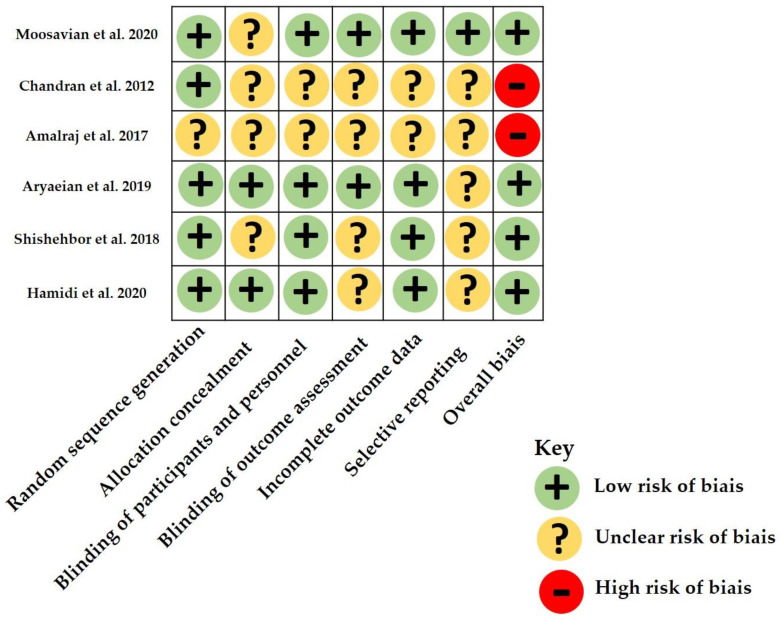
Summary of risk of bias according to the Cochrane Collaboration’s tool for the prevention trial.

The risk of bias in all six studies according to the Jadad score of randomized controlled trials and Cochrane’s tool is reported in Table S1 (Supplementary Materials) and Figure 2, respectively. All selected studies but one were double-blind randomized controlled trials. Chandran’s and Amalraj’s studies were considered at high risk of bias. Four of the six studies had a Jadad score of 4 or more. Two studies did not report the used randomization sequence.

### 3.4. Results of Individual Studies

Outcomes of each study are summarized in Table 3 according to the type of spice supplementation.

#### 3.4.1. Garlic Supplementation in Rheumatoid Arthritis

One double-blind randomized controlled trial by Moosavian et al. with a low risk of bias assessed the effect of a garlic supplementation (1000 mg garlic powder tablets equivalent to 2.5 g of fresh garlic, i.e., half of a garlic clove) on RA activity in 70 patients (35 per group) [11,12]. There was no significant difference between the two groups for DMARDS and corticosteroids. There was a significant decrease in the garlic group compared to the placebo group regarding DAS-28 ESR, tender joint count (TJC), VAS pain, and CRP. The reduction in VAS was little and not clinically pertinent (−9.11/100 in the garlic group vs. +1.35/100 in the placebo group, *p* < 0.001). Yet, the mean variation of DAS-28 ESR in the garlic group (−0.8) corresponded to a moderate EULAR response. There was no difference between the group regarding SJC, HAQ score, and ESR. However, the baseline SJC was very low (<2).

#### 3.4.2. Curcumin Supplementation in Rheumatoid Arthritis

Two randomized controlled trials (one double-blind and one single-blind) with a high risk of bias assessed the effect of curcumin supplementation (250 mg and 500 mg twice daily for one study and 500 mg twice daily for the other one, which represents between half and one teaspoon) on RA activity [13,14]. In these two trials, patients were excluded if they were treated with csDMARDs or bDMARDS. Chandran et al. assessed the effects of curcumin supplementation, the combination curcumin/diclofenac, and diclofenac in 45 patients (15 per group) [13]. DAS-28 ESR, SJC, TJC, VAS pain, and VAS activity decreased significantly in the three groups (curcumin, curcumin/diclofenac, and diclofenac), whereas CRP decrease significantly only in the curcumin group. The mean variation in DAS-28 ESR in the three groups corresponded to a high EULAR response. However, there was no statistical comparison between the three groups. Amalraj et al. assessed the effects of two dosages of curcumin supplementation (250 mg and 500 mg twice daily) in 36 patients (12 per group) [14]. DAS 28, SJC, TJC, VAS pain, CRP, and CRP decreased significantly in the curcumin groups, whereas the number of patients with ACR20 response increased significantly in the three groups. The mean variation of DAS-28 ESR in the curcumin groups corresponded to a high EULAR response. As for the previous study, there was no statistical comparison among the three groups.

#### 3.4.3. Ginger Supplementation in Rheumatoid Arthritis

One double-blind randomized controlled trial by Aryeian et al. with a low risk of bias assessed the effect of a ginger supplementation (750 mg twice daily, equivalent to one teaspoon) on RA activity in 63 patients [15,16]. There were significant decreases in the ginger group compared to the placebo group regarding DAS-28 ESR and CRP. Variations in clinical activity (DAS-28 ESR) were important in the treated group, with a mean variation corresponding to a high EULAR response.

#### 3.4.4. Cinnamon Supplementation in Rheumatoid Arthritis

One double-blind randomized controlled trial by Shishehbor et al. including 36 patients (18 per group) with a low risk of bias assessed the effect of a cinnamon supplementation (1000 mg twice daily, equivalent to two teaspoons) on RA activity [17]. There was a significant decrease in the ginger group compared to the placebo group regarding DAS-28, SJC, TJC, VAS pain, and CRP. Variations in DAS-28 were important in the treated group, with a mean variation corresponding to a high EULAR response. On the contrary, there was no significant difference between the two groups regarding ESR.

#### 3.4.5. Saffron Supplementation in Rheumatoid Arthritis

One double-blind randomized controlled trial by Hamidi et al. with a low risk of bias assessed the effect of a saffron supplementation (100 mg daily) on RA activity in 66 patients [18]. There was a significant decrease in the ginger group compared to the placebo group regarding DAS-28 ESR, SJC, VAS pain, and ESR. On the contrary, there was no significant difference between the two groups regarding TJC, morning stiffness, and CRP. The mean variation in DAS-28 corresponded to a moderate EULAR response.

## 4. Discussion

This is the first systematic literature review to focus on spice supplementation studies in RA. A previous systematic literature review on the efficacy of curcumin for alleviating the symptoms of joint arthritis included only one study with RA patients (Chandran et al. [13]). The seven other studies focused on patients with osteoarthritis [19]. All the studies included were published between 2017 and 2020, except for one published in 2012. This shows the growing interest of the potential benefit of spices for treating inflammatory diseases including RA. Spices have been used in several countries such as India, Thailand, and China, as well as several countries in Africa, for millennial flavoring, coloring, and preserving food, in addition to medicinal and religious purposes [2]. For example, curcumin is used during wedding and other religious ceremonies and recommended against poisoned food in Ayurvedic medicine [3]. Spices have been studied in multiple pathologies such as cancer, cardiovascular, gastrointestinal, neurodegenerative, metabolic, and infectious diseases due to their neuroprotection, antioxidant, anti-inflammatory, antibacterial, antifungal, and anticancer activities [2].

Studies of garlic, ginger, cinnamon, and saffron supplementation resulted in improvements in a combination of both subjective measures (e.g., VAS pain) and objective measures (e.g., CRP) of the disease. The benefits may relate to reduced inflammation due to inhibiting the NF-κB pathway or cyclooxygenase (COX) activation [5,6,7].

Diallyl sulfide, a flavor compound from garlic, downregulates RAC-alpha serine/threonine-protein kinase (Akt1)/Transforming growth factor beta (TGF-β)-activated kinase-mediated NF-κB and mitogen-activated protein kinase (MAPK) signaling pathways in murine macrophage-like cells [5]. It also diminished *Porphyromonas gingivalis*, lipopolysaccharide-stimulated cytokine expression, and NF-κB activation in human gingival fibroblasts [20]. *Porphyromonas gingivalis* is a potent periodontal pathogen involved in RA [21]. Active compounds from garlic inhibited the expressions of NF-κB-dependant genes in mice with ultraviolet B-irradiated skin [22].

6-Shogaol, a compound of ginger, inhibited the phosphoinositide 3-kinase (PI3K)/Akt and NF-κB signaling pathways in human intestinal epithelial cells [23]. Extract of red ginger reduced paw edema in a rat adjuvant arthritis model [24]. Ginger oil caused a significant suppression of both paw and joint swelling in rats with chronic adjuvant arthritis [25]. In the study assessing ginger in RA patients, ginger treatment significantly decreased the expression of the T-bet gene. T-bet is the T helper cell 1 (Th1) cell transcription factor that induces the proliferation of Th1, an essential cell in autoimmune diseases [16].

Cinnamon reduced NF-κB transcriptional activity via the suppression of DNA-binding activity in macrophages [26], inhibited the release of Tumour Necrosis Factor alpha (TNF-α) from neutrophils, and reduced the gene expression of proinflammatory cytokines [27]. It also inhibited the development of mice paw edema induced by carrageenan [28].

Saffron inhibits cyclooxygenase 1 and cyclooxygenase 2 enzymes. It prevented the production of prostaglandin E in a dose-dependent manner [7]. It also showed an anti-inflammatory activity in formaldehyde-induced arthritis [7].

Studies on curcumin supplementation are difficult to interpret as they are low-quality trials, with no comparison between intervention and the control groups [13,18]. Thus, no conclusion could be drawn on its impact on RA activity. Moreover, an ethical issue should be highlighted in the design of these two studies, as patients in the control group did not receive the standard care for RA.

Tolerance was good in the six studies: stomach pain in one patient with garlic and saffron supplementation [11,12,18] and mild fever and throat infection in one patient with curcumin supplementation [13]. Clinical trials assessing saffron, curcumin, and garlic supplementation showed similar mild adverse events between control and experimental groups [20,29,30]. Cinnamon supplementation in other clinical trials was associated with gastrointestinal disorders and allergic reactions, which were self-limiting in the majority of cases [31]. Heartburn, nausea, diarrhea, abdominal pain, bloating, gas, and epigastric distress were reported in clinical trials using ginger supplementation [32].

There are also limitations to the reviewed studies that need to be addressed. First, all studies were based on only a small sample size. Second, the duration of the clinical trials was short (between 8 and 12 weeks). Furthermore, most of the studies did not specify primary and secondary outcomes. Sample size was not determined in two studies [13,14]. Several points limit the external validity. Patients included in two studies did not receive the standard care, i.e., any DMARDs, to treat their disease [13,14]. It is unreasonable to expect these supplements to surpass the effect of DMARD therapy and, at most, they should be considered as an experimental adjunct. In the four other studies [11,12,15,16,17,18], patients with active RA did not receive bDMARDs, which is not the standard care for RA in Western countries [33,34]. Moreover, the six studies were done in only two countries: India and Iran. There was no study in Caucasian or African populations. Furthermore, daily saffron supplementation may not be affordable for every patient as saffron is a very rare and expensive spice. Lastly, a meta-analysis of findings could not be conducted due to the small number of studies per spice (one or two). No ongoing clinical trial to assess the impact of spice supplementation on RA activity is registered at clinicaltrials.gov. Thus, no supplementary data will be provided in the near future.

In summary, according to the available evidence, garlic, ginger, cinnamon, and saffron supplementation could have a beneficial effect on RA activity, but the risk of bias in these studies is difficult to assess and data are too limited to recommend them in daily practice.

## Figures and Tables

**Table 1:** Baseline characteristics of patients.

Study	Country	Inclusion Criteria	Intervention				Controls			
			Age (Years)	Disease Duration (Years)	RF+	ACPA+	Age (Years)	Disease Duration (Years)	RF+	ACPA+
Moosavian, 2020 [9,10]	Iran	ACR/EULAR criteria, DAS-28 ESR > 3.2, treated with csDMARDs, not receiving NSAIDs, or bDMARDs	G: 51.22 ± 12.61	G: 6.60 ± 7.43	NR	NR	51.37 ± 11.04	6.68 ± 8.20	NR	NR
Chandran, 2012 [11]	India	ACR 1987, DAS-28 ESR > 5.1, not receiving NSAIDs, csDMARDs, or bDMARDs	C: 7.8 ± 8.60 C + D: 47 ± 16.22	NR	NR	NR	48.87 ± 10.78	NR	NR	NR
Amalraj, 2017 [12]	India	ACR/EULAR criteria, DAS-28 ESR > 5.1, CRP > 0.6 mg/dL or ESR > 28 mm/h, not receiving NSAIDs, csDMARDs, or bDMARDs	C 250 mg 36.7 ± 10.7 C 500 mg 38.3 ± 5.8	NR	NR	NR	39.6 ± 8.8	NR	NR	NR
Aryaeian, 2019 [13,14]	Iran	ACR/EULAR criteria, 2 year disease duration, treated with methotrexate, hydroxychloroquine, and prednisolone < 10 mg/day	Gi: 48.63 ± 2.38	Gi: 18.12 ± 4.13	NR	NR	46.67 ± 1.94	14.87 ± 4.13	NR	NR
Shishehbor, 2018 [15]	Iran	ACR/EULAR criteria, for at least 2 years, having active disease, treated with csDMARDs, not receiving NSAIDs or bDMARDs	Ci: 44.66 ± 11.22	6.27 ± 3.04	NR	NR	49.11 ± 7.45	5.00 ± 2.22	NR	NR
Hamidi, 2020 [16]	Iran	ACR/EULAR criteria, for at least 2 years, having active disease	S: 51.55 ± 8.26	S: 10.74 ± 5.66	NR	NR	51.80 ± 9.62	9.60 ± 5.13	NR	NR

Abbreviations: ACR: American College of Rheumatology; ACPA+: anti-citrullinated protein/peptide antibodies positive; bDMARDs: biologic disease-modifying antirheumatic drugs; csDMARDs: conventional synthetic disease-modifying antirheumatic drugs; C: curcumin; C + D: curcumin + diclofenac; Ci: cinnamon; DAS-28: disease activity score 28; ESR: erythrocyte sedimentation rate; EULAR: European League against Rheumatism; G: garlic; Gi: ginger; NR: not reported; NSAIDs: nonsteroidal anti-inflammatory drugs; RF+: rheumatoid factor positive; S: saffron;. Values are expressed as means ± standard deviation.

**Table 2:** Study characteristics.

				Intervention		Controls		Outcome	Outcome Measurement
Spice	Study	Design	Population	Type	*N*	Type	*N*		
Garlic	Moosavian, 2020 [11,12]	Double-blind RCT	70	1000 mg garlic powder tablets	35	Placebo	35	DAS-28 ESR, SJC, TJC, VAS Pain, HAQ score, CRP, ESR	8 weeks
				equivalent to 2.5 g of fresh garlic					
Curcumin	Chandran, 2012 [13]	Single-blind RCT	45 in 3 groups	Curcumin 500 mg twice a day and diclofenac 50 mg twice a day	15	Diclofenac 50 mg × 2/day	15	DAS-28 ESR, SJC, TJC, VAS pain, VAS activity, HAQ score, CRP, ESR	8 weeks
				Curcumin 500 mg twice a day	15				
Curcumin	Amalraj, 2017 [14]	Double-blind RCT	36 in 3 groups	Curcumin 250 mg twice a day	12	Placebo	12	ACR-20, DAS-28, SJC, TJC, VAS pain, CRP, ESR	12 weeks
				Curcumin 500 mg twice a day	12				
Ginger	Aryaeian, 2019 [15,16]	Double-blind RCT	63	Ginger powder 750 mg twice a day	33	Placebo	30	DAS-28 ESR, CRP	12 weeks
Cinnamon	Shishehbor, 2018 [17]	Double-blind RCT	36	Cinnamon 1 g twice a day	18	Placebo	18	DAS-28, SJC, TJC, VAS pain, ESR, CRP	8 weeks
Saffron	Hamidi, 2020 [18]	Double-blind RCT	66	Saffron 100 mg per day	33	Placebo	33	DAS-28 ESR, SJC, TJC, VAS pain, morning stiffness, CRP, ESR	12 weeks

Abbreviations: RCT: randomized controlled trial; DAS-28: disease activity score 28; VAS: visual analogue scale; ACR: American College of Rheumatology; HAQ: health assessment questionnaire; TJC: tender joint count; SJC: swollen joint count; ESR: erythrocyte sedimentation rate; CRP: C reactive protein.

**Table 3:** Study results sorted by spice supplementation.

Study	Outcome	Intervention	Controls	Between-Group Differences
		Baseline versus End of Treatment	Baseline versus End of Treatment	
				*p*-Value
Moosavian et al. [11,12]	DAS-28 ESR	G: 4.61 ± 0.92 vs. 3.80 ± 0.81 *	4.52 ± 0.78 vs. 4.45 ± 0.86	<0.001
	SJC	G: 1.92 ± 1.62 vs. 1.19 ± 1.40 *	1.74 ± 2.17 vs. 1.71 ± 2.34	0.117
	TJC	G: 6.74 ± 4.55 vs. 3.61 ± 4.04 *	5.57 ± 3.97 vs. 5.55 ± 4.5	<0.001
	VAS Pain (mm)	G: 68.46 ± 14.80 vs. 59.35 ± 13.30 *	70.54 ± 16.66 vs. 69.19 ± 18.40	<0.001
	HAQ score	G: NR	NR	0.23
	CRP (mg/L)	G: 13.44 ± 13.76 vs. 8.62 ± 10.58 *	13.57 ± 14.04 vs. 14.23 ± 16.22	0.018
	ESR (mm/h)	G: 23.63 ± 13.82 vs. 19.03 ± 12.94	20.10 ± 11.74 vs. 20.74 ± 13.26	0.134
Chandran et al. [6]	DAS-28 ESR	C + D: 6.44 ± 0.51 vs. 3.58 ± 0.71 *	6.72 ± 0.87 vs. 3.89 ± 1.43 *	NR
		C: 6.40 ± 0.73 vs. 3.55 ± 0.73 *		NR
	SJC	C + D: 11.5 vs. 0.42 *	16.6 vs. 1.83 *	NR
		C: 12.15 vs. 0.36 *		NR
	TJC	C + D: 16.67 vs. 2.75 *	18.2 vs. 5.67 *	NR
		C: 18.64 vs. 3.14 *		NR
	VAS Pain (mm)	C + D: 77.25 ± 9.65 vs. 34.29 ± 26.75 *	78.25 ± 11.25 vs. 39.17 ± 20.1 *	NR
		C: 68.57 ± 17.14 vs. 27.5 ± 9.35 *		NR
	VAS Activity (mm)	C + D: 78.75 40.83 *	77.5 vs. 42.08 *	NR
		C: 83.93 vs. 30.7 *		NR
	HAQ score	C + D: 3.95 vs. 1.53 *	3.79 vs. 1.51 *	NR
		C: 4.41 vs. 1.0 *		NR
	CRP (mg/L)	C + D: 9.11 ± 9.93 vs. 6.66 ± 6.87 *	3.3 ± 2.4 vs. 3.35 ± 2.5	NR
		C: 5.34 ± 4.12 vs. 2.56 ± 1.8		NR
	ESR (mm/h)	C + D: 28.75 ± 20.09 vs. 24.92 ± 22.6	27.08 ± 17.1 vs. 24.75 ± 13.5	NR
		C: 28 ± 23.7 vs. 24.86 ± 17.7		NR
Amalraj et al. [7]	DAS-28	C 250 mg: 4.51 ± 0.64 vs. 2.14 ± 0.16 *	3.53 ± 0.47 vs. 3.53 ± 0.47	NR
		C 500 mg: 5.29 ± 0.54 vs. 1.80 ± 0.36 *		NR
	ACR-20	C 250 mg: 19.33 ± 2.81 vs. 65.17 ± 10.67 *	14.75 ± 6.58 vs. 14.75 ± 6.58 *	NR
		C 500 mg: 16.50 ± 3.78 vs. 67.83 ± 8.60 *		NR
	SJC	C 250 mg: 14.42 ± 1.68 vs. 2.83 ± 0.83 *	11.08 ± 2.23 vs. 10.67 ± 1.97	NR
		C 500 mg: 17.00 ± 1.35 vs. 2.58 ± 0.67 *		NR
	TJC	C 250 mg: 13.33 ± 3.17 vs. 2.92 ± 0.67 *	9.50 ± 3.23 vs. 9.92 ± 1.93	NR
		C 500 mg: 16.67 ± 1.92 vs. 2.00 ± 0.74 *		NR
	VAS pain (cm)	C 250 mg: 7.01 ± 0.86 vs. 2.63 ± 0.74 *	6.61 ± 0.73 vs. 6.84 ± 0.63	NR
		C 500 mg: 7.99 ± 0.71 vs. 2.21 ± 0.45 *		NR
	CRP (mg/dL)	C 250 mg: 0.97 ± 0.15 vs. 0.68 ± 0.10 *	0.97 ± 0.15 vs. 1.08 ± 0.15	NR
		C 500 mg: 1.21 ± 0.18 vs. 0.59 ± 0.08 *		NR
	ESR (mm/h)	C 250 mg: 175.9 ± 12.9 vs. 21.0 ± 4.8 *	180.2 ± 12.4 vs. 126.9 ± 17.3	NR
		C 500 mg: 181.7 ± 4.8 vs. 21.2 ± 2.9 *		NR
Aryaeian et al. [8,9]	DAS-28-ESR	Gi: 4.73 ± 0.27 vs. 3.44 ± 0.30 *	4.51 ± 0.27 vs. 4.30 ± 0.33	<0.001
	CRP (mg/dL)	Gi: 13.50 ± 3.45 vs. 7.62 ± 5.1 *	13.01 ± 2.25 vs. 16.39 ± 9.6	0.044
Shishehbor et al. [10]	DAS-28	Ci: 6.04 ± 0.52 vs. 3.92 ± 0.52 *	5.35 ± 0.76 vs. 5.64 ± 0.66	<0.001
	SJC	Ci: 8.44 ± 2.33 vs. 1.38 ± 0.97 *	7.16 ± 2.23 vs. 7.66 ± 2.08	<0.0001
	TJC	Ci: 11.44 ± 2.52 vs. 2.77 ± 1.47 *	10.05 ± 2.66 vs. 10.05 ± 3.09	<0.001
	VAS pain (cm)	Ci: 68.88 ± 14.30 vs. 43.88 ± 12.89 *	54.72 ± 16.58 vs. 58.05 ± 18.24	<0.001
	CRP (mg/L)	Ci: 35.33 ± 10.08 vs. 24.61 ± 10.29 *	27 ± 12.92 vs. 32.50 ± 13.15 *	<0.001
	ESR (mm/h)	Ci: 32.88 ± 13.31 vs. 23.66 ± 12.98 *	25.16 ± 17.44 vs. 27.83 ± 17.74	0.42
Hamidi et al. [11]	DAS-28 ESR	S: 5.09 ± 1.10 vs. 4.33 ± 0.94 *	4.92 ± 1.09 vs. 5.19 ± 0.65	<0.001
	SJC	S: 6.26 ± 3.63 vs. 4.13 ± 2.47 *	7.07 ± 3.83 vs. 7.70 ± 2.54	≤0.001
	TJC	S: 5.23 ± 3.27 vs. 3.84 ± 2.70 *	4.53 ± 2.86 vs. 4.63 ± 2.73	0.259
	VAS pain (mm)	S: 60.97 ± 21.19 vs. 42.58 ± 15.69 *	52.33 ± 22.99 vs. 50.00 ± 21.81 *	≤0.001
	Morning stiffness: 1–3 h	S: 10 (32.30%) vs. 6 (19.40%)	5 (16.70%) vs. 6 (20.00%)	0.975
	CRP (mg/L)	S: 12.00 ± 7.40 vs. 8.82 ± 7.93 *	12.00 ± 12.84 vs. 14.56 ± 21.03	0.200
	ESR (mm/h)	S: 29.94 ± 17.40 vs. 24.06 ± 12.66 *	30.20 ± 28.19 vs. 32.00 ± 14.75	0.028

Abbreviations: ACR: American College of Rheumatology; DAS-28: disease activity score 28; VAS: visual analogue scale for pain; C: curcumin; C + D: curcumin + diclofenac; Ci: cinnamon; G: garlic; Gi: ginger; HAQ: health assessment questionnaire; NR: not reported; S: saffron; SJC: swollen joint count; TJC: tender joint count. Data are presented as the mean and standard deviation or number of patients with the proportion in percentages; * significant difference within the group.

## Supplementary Materials

The following are available online at https://www.mdpi.com/2072-6643/12/12/3800/s1, Table S1: Quality assessment based on Jadad score of randomized controlled trials reviewed.
