# Supplementary material for: Efficacy of Spice Supplementation in Rheumatoid Arthritis: A Systematic Literature Review
Source: Nutrients. 2020 Dec 11;12(12):3800. doi: 10.3390/nu12123800 (PMC7764619; doi:10.3390/nu12123800)
Supplement: Supplementary file 1 [file nutrients-12-03800-s001.pdf]

nutrients

Table S1. Quality assessment based on Jadad score of randomized controlled trials reviewed.

Study	Randomization	Blinding	Account of All Patients	Total
Moosavian et al. [11,12]	**2**	**2**	**1**	**5**
Chandran et al. [13]	**2**	**0**	**0**	**2**
Amalraj et al. [14]	**1**	**1**	**0**	**2**
Aryaeian et al. [13.14]	**2**	**2**	**0**	**4**
Shishehbor et al. [17]	**2**	**1**	**1**	**4**
Hamidi et al. [18]	**2**	**2**	**1**	**5**
